# HCN2 Promotes BGN Transcription via REST to Regulate Ferroptosis and Tumor Progression in Bladder Cancer

**DOI:** 10.3390/ijms27083433

**Published:** 2026-04-11

**Authors:** Yudong Cao, Jinchao Ma, Xingxing Tang, Yushuang Cui, Xiao Yang, Yongpeng Ji, Ruijian You, Chen Lin, Shuo Wang, Peng Du

**Affiliations:** Key Laboratory of Carcinogenesis and Translational Research (Ministry of Education/Beijing), Department of Urology, Peking University Cancer Hospital & Institute, Beijing 100142, China

**Keywords:** bladder cancer, HCN2, BGN, transcriptional regulation, ferroptosis

## Abstract

Bladder cancer is one of the most common malignancies of the urinary system. Identifying new potential therapeutic targets and exploring molecular mechanisms are crucial for improving treatment and prognosis. The hyperpolarization-activated cyclic nucleotide-gated (HCN) channel 2, known to play a key role in various physiological and pathological processes, has an unclear function and mechanism of action in bladder cancer. We employed bioinformatics analysis and immunohistochemistry to assess the role of HCN2 in bladder cancer, integrating in vitro and in vivo models to evaluate the impact of HCN2 on cell behavior. Molecular interactions were characterized using immunoprecipitation, chromatin immunoprecipitation, and dual-luciferase reporter assays. Our investigation revealed a significant upregulation of HCN2 in bladder cancer tissues, which was predictive of a poorer clinical outcome. Functionally, HCN2 knockdown in bladder cancer impeded cell proliferation, induced apoptosis, and curtailed migration and invasion. Mechanistically, the overexpression of HCN2 contributed to the translocation of the REST transcription factor into the nucleus and facilitated its binding to the BGN promoter for transcriptional activation of its expression. This regulatory mechanism was shown to suppress ferroptosis, a form of regulated cell death, thereby enhancing the proliferative and tumorigenesis of bladder cancer cells. This study uncovers the novel mechanism by which HCN2 regulates ferroptosis via the REST-BGN axis, affecting bladder cancer cell behavior, and provides new perspectives and strategies for future clinical treatment.

## 1. Introduction

Bladder cancer is a common malignant tumor of the urinary system. According to Globocan 2022 statistics, nearly 570,000 new cases of bladder cancer were diagnosed globally in 2020, with over 210,000 deaths, posing a significant threat to global public health [1]. Despite continuous optimization of treatment strategies, including surgery, radiotherapy, systemic therapies such as chemotherapy, immunotherapy, and antibody-drug conjugates (ADCs), the high mortality rate of locally advanced or metastatic bladder cancer remains unresolved [2,3,4]. This situation underscores the urgent need for the development of new treatment strategies. Targeted therapy, which selectively inhibits tumor growth by targeting specific molecular changes in cancer cells, has become a promising approach for treating various malignancies [5]. For example, FGFR 3-targeted therapy has shown good efficacy in second-line treatment of metastatic urothelial carcinoma [6]. However, the efficacy of targeted therapies is often limited by factors such as drug resistance and off-target effects, necessitating a deeper understanding of the molecular landscape of bladder cancer to identify novel and effective therapeutic targets.

In recent years, the role of genetic alterations in the development of bladder cancer has become increasingly recognized [7]. Among these genetic changes, the potential involvement of the hyperpolarization-activated cyclic nucleotide-gated 2 (HCN2) channel in the pathogenesis of bladder cancer has garnered significant interest. The HCN2 channel is known to play an important role in regulating cellular excitability and pacemaker activity, being associated with arrhythmias and pain, and potentially serving as a target for pain treatment [8,9,10]. Emerging evidence suggests that the HCN2 channel may interact with signaling pathways critical for cancer cell growth and survival. For instance, the HCN2 channel has been shown to regulate the activity of various protein kinases, including protein kinase A (PKA) and protein kinase C (PKC) [11], which play key roles in cancer cell proliferation and migration. By regulating these signaling pathways, the HCN2 channel may promote tumor progression and metastasis. HCN2 is also involved in apoptosis; in lung cancer cells, dephosphorylation of the Thr549 site within the HCN2 C-terminal domain can trigger apoptosis mediated by the apoptosis-inducing factor [12]. Moreover, Ling S et al. found that compared to normal salivary gland tissue, the HCN2 promoter is hypomethylated in primary adenoid cystic carcinoma tumors, and HCN2 promoter hypomethylation serves as a biomarker for adenoid cystic carcinoma [13]. However, the molecular mechanism by which the HCN2 channel is involved in bladder cancer remains unclear.

In this study, we aimed to comprehensively explore the molecular role and regulatory mechanisms of the HCN2 channel in bladder cancer. Specifically, we analyzed the expression of the HCN2 channel in bladder cancer tissues and cell lines. Additionally, we modulated HCN2 expressions and investigated its effects on cancer cell proliferation, migration, and invasion. By revealing the role and mechanisms of HCN2 in bladder cancer, this study provides new insights into the pathogenesis of bladder cancer and potentially offers new strategies for the development of targeted precision therapies for bladder cancer patients.

## 2. Results

### 2.1. HCN2 Expression in Bladder Cancer and Its Association with Pathological Features and Survival

**Figure 1:**
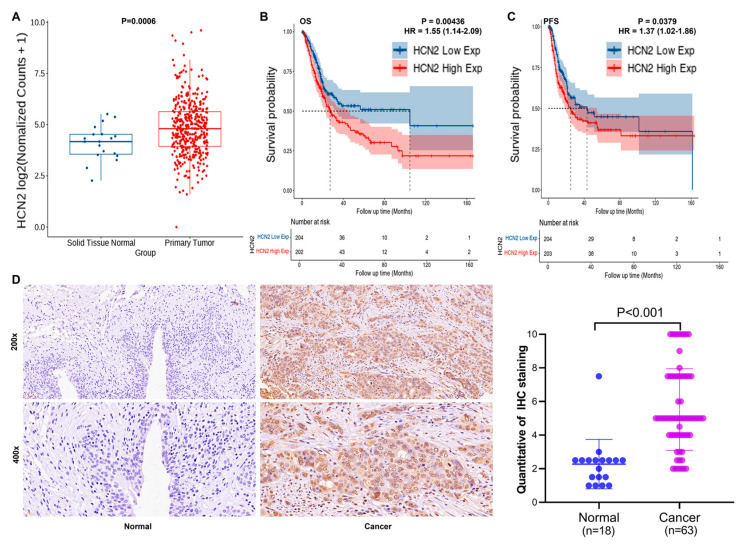
HCN2 expression is upregulated in bladder cancer and associated with poor prognosis. (**A**) A box plot was used to visualize the differential expression of HCN2 between bladder urothelial carcinoma and normal urothelial tissues, based on RNA-seq data retrieved from The Cancer Genome Atlas (TCGA) database. (**B**,**C**) The median expression level of HCN2 across all bladder urothelial carcinoma samples was used as the cutoff value to dichotomize patients into high- and low-expression groups. Overall survival (OS; (**B**)) and Progression-free survival (PFS; (**C**)) differences between the two groups were assessed using the log-rank test. (**D**) Representative immunohistochemical staining of HCN2 in bladder cancer tissues and cancer-adjacent tissues.

Bioinformatics analysis of TCGA database samples revealed that HCN2 is significantly upregulated in bladder urothelial carcinoma compared to normal tissues (*p* = 0.0006), with a fold-change difference of 1.3 between cancerous and normal tissues (Figure 1A). Using the median HCN2 expression in bladder urothelial carcinoma as a threshold, we divided the samples into high-expression and low-expression groups. Analysis of overall survival (OS) and progression-free survival (PFS) showed that higher HCN2 expression was associated with significantly shorter OS (median OS for high expression group = 27.06 (22.52–44.32) months, Log-rank_P = 0.00436, HR = 1.55 (1.14–2.09)) compared to the low expression group (median OS = 104.65 (33.04-NA) months). Similarly, high HCN2 expression correlated with shorter PFS (median PFS for high expression group = 24.92 (17.98–54.28) months, Log-rank_P = 0.0379, HR = 1.37 (1.02–1.86)) compared to the low expression group (median PFS = 43.2 (23.18-NA) months) (Figure 1B,C). These findings suggest that high HCN2 expression may be associated with poor prognosis in bladder cancer.

To validate these bioinformatics results, we constructed tissue microarrays from clinical samples, including normal tissues (*n* = 18) and tumor tissues (*n* = 63), and performed immunohistochemistry (IHC) staining. Quantitative analysis of HCN2 expression in bladder cancer and normal tissues (Table S1) and staining images (Figure 1D) confirmed that HCN2 is abnormally elevated in bladder cancer.

We further explored the relationship between HCN2 expression levels and clinicopathological characteristics of bladder cancer using Mann–Whitney U analysis. HCN2 expression was found to be significantly positively correlated with T stage and clinical stage (Table S2). Spearman rank correlation analysis also supported these results, indicating that HCN2 expression increases with the severity of tumor malignancy (Table S3). Collectively, these findings suggest that HCN2 may be associated with bladder cancer development, progression, and prognosis, potentially serving as a therapeutic target.

### 2.2. HCN2 Knockdown Inhibits Bladder Cancer Cell Progression

**Figure 2:**
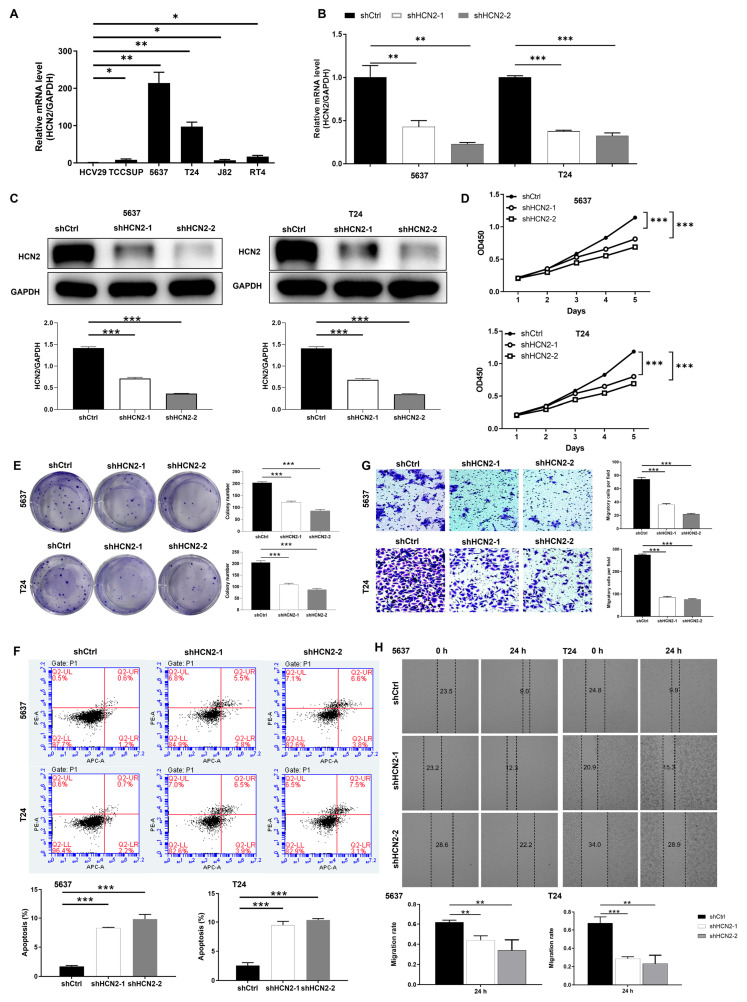
Knockdown of HCN2 inhibits bladder cancer cell progression. (**A**) Relative HCN2 mRNA expression in bladder cancer cell lines (TCCSUP, 5637, T24, J82, RT4) compared to normal bladder epithelial cells (HCV29) by RT-qPCR. Data are mean ± SD from three independent experiments; *p* < 0.05 vs. HCV29 (Student’s *t*-test). (**B**) RT-qPCR analysis of HCN2 mRNA levels in shCtrl, shHCN2-1, and shHCN2-2 5637/T24 cells. Data are mean ± SD from three independent experiments. (**C**) Western blot confirmation of HCN2 protein knockdown in 5637/T24 cells (GAPDH = loading control). (**D**) CCK-8 assay of cell proliferation in shCtrl, shHCN2-1, and shHCN2-2 5637/T24 cells. Data are mean ± SD from three independent experiments. (**E**) Colony formation assay of 5637/T24 cells after HCN2 knockdown. Data are mean ± SD from three independent experiments. (**F**) Flow cytometry analysis of apoptosis in 5637 and T24 cells using Annexin V-FITC/PI dual staining. Cells were categorized into four quadrants: Q_4_ (Annexin V^−^/PI^−^, viable), Q_3_ (Annexin V^+^/PI^−^, early apoptotic), Q_2_ (Annexin V^+^/PI^+^, late apoptotic/necrotic), and Q_1_ (Annexin V^−^/PI^+^, necrotic). Apoptosis rate = Q_2_ + Q_3_. Data are mean ± SD from three independent experiments. *p* < 0.001 vs. shCtrl (Student’s *t*-test). (**G**) Wound healing assay of 5637/T24 cell migration after HCN2 knockdown. Data are mean ± SD from three independent experiments. (**H**) Transwell invasion assay of 5637/T24 cells after HCN2 knockdown. Data are mean ± SD from three independent experiments. Statistical analyses: Student’s *t*-test (two groups) or one-way ANOVA (multiple groups); * *p* < 0.05, ** *p* < 0.01, *** *p* < 0.001.

To investigate the effects of HCN2 on bladder cancer cell phenotype and biological function, we conducted a series of experiments at the cellular level, with all assays independently repeated three times (biological replicates, *n* = 3). First, we quantified endogenous HCN2 expression levels in bladder cancer cell lines (TCCSUP, 5637, T24, J82, and RT4) using quantitative real-time PCR (qPCR). Compared to normal bladder epithelial cells (HCV29), HCN2 expression was significantly upregulated in the cancer cell lines (*p* < 0.05), with particularly high expression in the 5637 and T24 cell lines (Figure 2A).

We then selected the shHCN2-1 and shHCN2-2 sequences based on target screening results (Figure S1A) and constructed HCN2-knockdown bladder cancer cell models. Successful establishment of dual-target HCN2 knockdown in 5637 and T24 cells was confirmed by infection efficiency (Figure S1B, observed under green fluorescent protein microscopy) and knockdown efficiency (mRNA: Figure 2B; protein: Figure 2C). CCK-8 assays showed that HCN2 knockdown inhibited bladder cancer cell proliferation (Figure 2D; mean ± SD from three independent experiments). Additionally, the ability of HCN2-knockdown cells to form colonies was significantly reduced (Figure 2E).

To explore the impact of HCN2 knockdown on cell apoptosis, we performed Annexin V-FITC/PI dual staining followed by flow cytometry (Figure 2F; apoptosis rates = Q_2_ + Q_3_). Compared with the shCtrl group, both shHCN2-1 and shHCN2-2 significantly elevated the apoptosis rate in 5637 and T24 cells. These data indicate that HCN2 knockdown drives bladder cancer cells toward apoptotic cell death (not necrosis), supporting a primary role of apoptosis in this process.

Scratch assays and Transwell assays demonstrated that HCN2 knockdown significantly inhibited the migration and invasion of bladder cancer cells (Figure 2G,H). Collectively, these data from three independent experiments suggest that HCN2 plays a key role in driving the malignant progression of bladder cancer.

### 2.3. Molecular Mechanism of HCN2 Promoting BGN Transcription via REST

**Figure 3:**
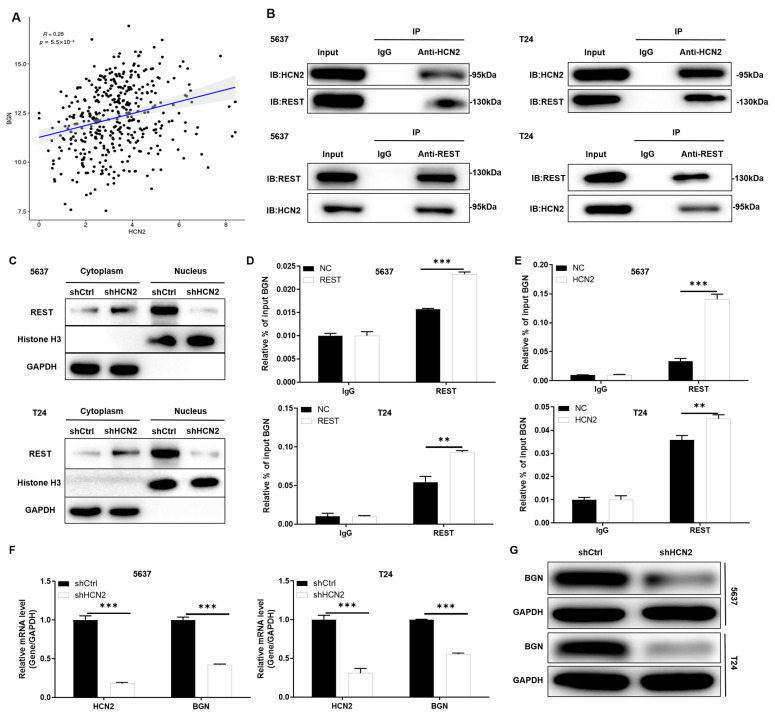
Molecular mechanism of HCN2 promoting BGN transcription via REST. (**A**) Positive correlation between HCN2 and BGN expression in bladder cancer as determined by Pearson Correlation Analysis using TCGA Database. (**B**) The Co-IP experiment was performed to verify the interaction between HCN2 and REST in 5637 and T24 cells. (**C**) HCN2 knockdown reduces REST nuclear translocation as determined by nuclear-cytoplasmic fractionation Western blotting analysis. (**D**) Transcriptional regulation of BGN by REST overexpression in 5637 and T24 cells as determined by CHIP-qPCR. (**E**) Transcriptional regulation of BGN by HCN2 overexpression in 5637 and T24 cells as determined by CHIP-qPCR. (**F**,**G**) Assessment of BGN mRNA and protein expression following HCN2 knockdown in 5637 (**F**) and T24 (**G**) cells via qPCR and Western blotting analysis. Data are presented as mean ± SD from three independent experiments. Statistical analyses: Student’s *t*-test (two groups) or one-way ANOVA (multiple groups); ** *p* < 0.01, *** *p* < 0.001.

To elucidate the molecular mechanism by which HCN2 regulates bladder cancer progression, we conducted bioinformatics exploration. Based on the STRING database, we identified REST as a candidate molecule interacting with HCN2. Using the GTRD database, we predicted BGN as a downstream target of the transcription factor REST. Pearson correlation analysis of bladder cancer samples from the TCGA database showed a positive correlation between HCN2 and BGN expression (Figure 3A). Based on these predictions, we hypothesized that HCN2 might regulate BGN transcription through REST.

Our hypothesis was substantiated by co-immunoprecipitation assays, which confirmed a physical interaction between HCN2 and REST proteins (Figure 3B). Additionally, Western blot analysis following nuclear-cytoplasmic separation revealed that HCN2 knockdown reduced REST nuclear translocation (Figure 3C). CHIP-qPCR experiments in REST-overexpressing and control 5637 and T24 cells validated that REST could bind to the BGN promoter, thereby exerting transcriptional regulation (Figure 3D).

Building upon these findings, in REST-overexpressing and control 5637 and T24 cells, CHIP-qPCR analysis confirmed that HCN2 overexpression promoted REST binding to the BGN promoter, leading to transcriptional regulation of BGN (Figure 3E). Ultimately, qPCR and WB experiments confirmed that HCN2 knockdown downregulated BGN mRNA and protein expression (Figure 3F,G). These results suggest that in bladder cancer, HCN2 upregulates BGN expression by promoting its transcription via REST.

### 2.4. HCN2 Drives Bladder Cancer Progression by Inhibiting Ferroptosis via BGN

**Figure 4:**
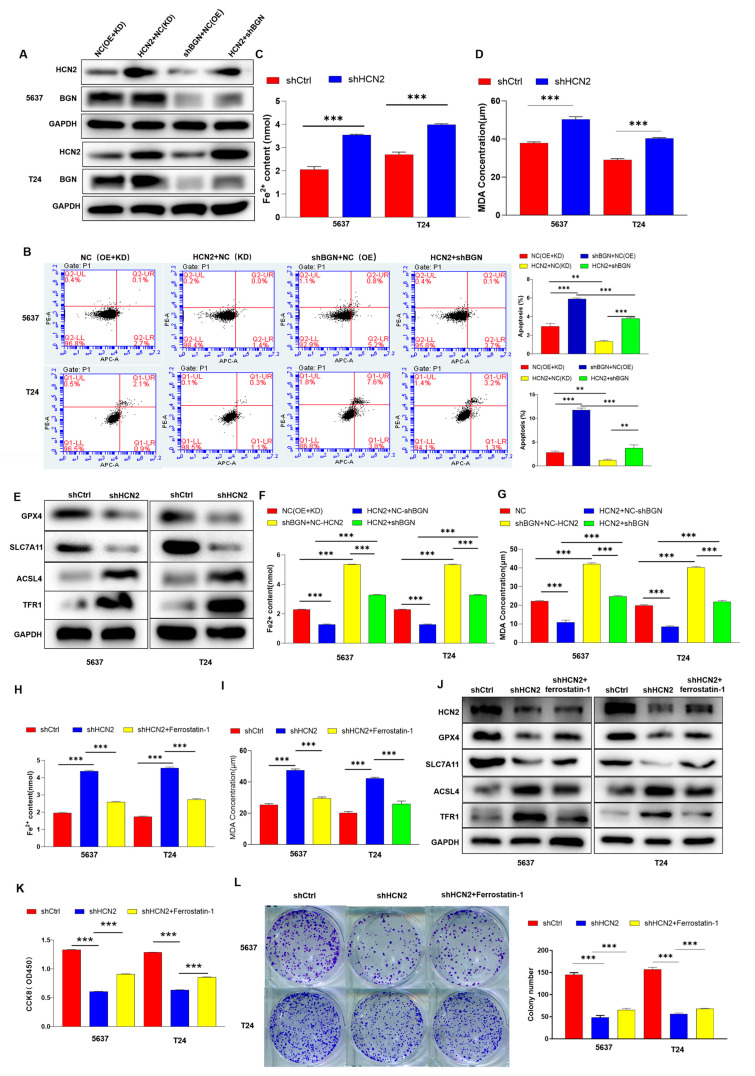
HCN2 promotes bladder cancer progression by inhibiting ferroptosis through BGN. (**A**) Western blot analysis of HCN2, BGN, and GAPDH (loading control) in 5637 and T24 cells after establishing HCN2 knockdown (shHCN2) and BGN overexpression (BGN) cell models. NC: Negative control (scramble shRNA for HCN2 knockdown or empty vector for BGN overexpression). (**B**) Flow cytometry analysis of apoptosis in 5637 and T24 cells. Groups: NC (OE/KD): Cells with negative control for overexpression/knockdown experiments (empty vector for HCN2 overexpression, scramble shRNA for HCN2 knockdown). HCN2+NC (KD): HCN2 knockdown cells transfected with negative control for BGN overexpression. shBGN+NC (OE): BGN overexpression cells transfected with negative control for HCN2 knockdown. HCN2+shBGN: HCN2 overexpression cells with BGN knockdown. Annexin V^+^/PI^−^: Apoptotic cells (phosphatidylserine externalization, membrane integrity preserved in early apoptosis); PI^+^: Necrotic cells (severe membrane damage). (**C**,**E**) Ferroptosis assessment in 5637 and T24 cells. (**C**) Intracellular Fe^2+^ content; (**D**) MDA levels; (**E**) Western blot analysis of ferroptosis-related proteins (GPX4, SLC7A11, ACSL4, TFR1) and GAPDH (loading control). (**F**,**G**) Partial rescue of ferroptosis by BGN overexpression in HCN2 knockdown cells. (**F**) Fe^2+^ content; (**G**) MDA levels. Groups: NC(OE+KD): Cells with negative control for both overexpression and knockdown. HCN2+NC(OE): HCN2 overexpression cells with negative control for BGN knockdown. HCN2+KD(KD): HCN2 knockdown cells with negative control for BGN overexpression. HCN2+shBGN: HCN2 overexpression cells with BGN knockdown. (**H**–**L**) Effects of ferrostatin-1 (ferroptosis inhibitor) on HCN2 knockdown-mediated phenotypes. (**H**) Fe^2+^ content; (**I**) MDA levels; (**J**) Western blot of ferroptosis-related proteins; (**K**) Cell proliferation (CCK-8 assay); (**L**) Colony formation ability. Groups: shCtrl: Negative control shRNA; shHCN2: HCN2 knockdown; shHCN2+ferrostatin-1: HCN2 knockdown cells treated with ferrostatin-1 (1 μM, pre-incubated 2 h before HCN2 knockdown). Data are presented as mean ± SD from three independent experiments. Statistical analyses were performed using one-way ANOVA; ** *p* < 0.01, *** *p* < 0.001.

We successfully constructed HCN2 knockdown and BGN overexpressing 5637 and T24 cell lines (Figure 4A) and conducted loss-of-function and gain-of-function experiments. Flow cytometry analysis showed that BGN overexpression partially rescued HCN2 knockdown-induced apoptosis in these bladder cancer cells (Figure 4B). Notably, while apoptosis and ferroptosis are distinct cell death modalities (apoptosis is characterized by phosphatidylserine externalization and maintained plasma membrane integrity in early stages, whereas ferroptosis features lipid peroxidation and membrane damage driven by iron-dependent lipid reactive oxygen species [14]), BGN’s rescuing effect on apoptosis aligns with its previously reported role in inhibiting ferroptosis in gastric cancer [15]. This suggests BGN may act as a key regulator of multiple cell survival pathways in bladder cancer, coordinating the suppression of both apoptotic and ferroptosis cell death to promote tumor progression. In this study, we demonstrated that BGN knockdown promotes ferroptosis in bladder cancer cells by measuring Fe^2+^ content (Figure S1C), MDA levels (Figure S1D), and ferroptosis-related protein expression (GPX4, SLC7A11, ACSL4, TFR1) (Figure S1E).

Given the potential synergistic role of HCN2 and BGN in bladder cancer cells, we hypothesized that HCN2 might influence ferroptosis in a BGN-dependent manner, thereby regulating bladder cancer cell progression. Our results demonstrated that HCN2 knockdown significantly promoted ferroptosis in the 5637 and T24 bladder cancer cell lines, as evidenced by increased iron content (Figure 4C), elevated MDA levels (Figure 4D), and abnormal expression of ferroptosis-related proteins (Figure 4E). Moreover, BGN knockdown partially rescued HCN2 overexpression-induced ferroptosis, as indicated by changes in iron content (Figure 4F) and MDA levels (Figure 4G). Thus, HCN2 appears to influence ferroptosis in a BGN-dependent manner.

Treatment of HCN2-knockdown 5637 and T24 cell lines with the ferroptosis inhibitor ferrostatin-1 demonstrated that this treatment could attenuate the effects of HCN2 knockdown on ferroptosis, proliferation, and colony formation in bladder cancer cells (Figure 4H–L). While ferrostatin-1 treatment reduced Fe^2+^ accumulation (Figure 4H) and MDA levels (Figure 4I) in HCN2-knockdown cells, these parameters did not fully return to control levels, indicating a partial rescue. Similarly, ferrostatin-1 restored cell proliferation (Figure 4K) and colony-forming ability (Figure 4L) but did not completely rescue the inhibitory effects of HCN2 knockdown, consistent with the partial nature of ferroptosis inhibition observed in biochemical assays.

### 2.5. In Vivo Validation of HCN2 Promoting Bladder Cancer Progression via Ferroptosis

**Figure 5:**
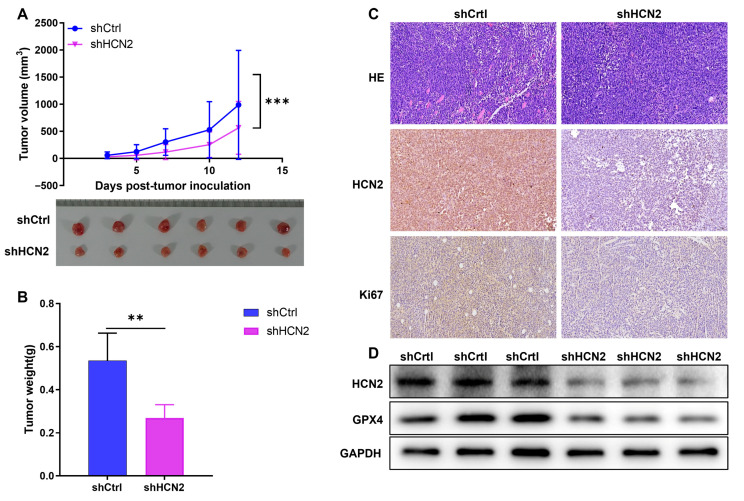
Loss of HCN2 inhibits bladder cancer growth in vivo. (**A**) **Top**: Tumor growth curves of mice subcutaneously injected with shCtrl or shHCN2 T24 cells (*n* = 6/group, three independent experiments). Error bars = mean ± SD. **Bottom**: Representative images of excised tumors. (**B**) Mean tumor weight of shCtrl vs. shHCN2 groups. Data are mean ± SD; *p* < 0.05 (Student’s *t*-test). (**C**) HE staining (200×) and IHC staining (200×) of tumor sections for Ki-67 (proliferation marker) and HCN2 expression. (**D**) Western blot analysis of HCN2 and GPX4 (ferroptosis marker) protein levels in shCtrl vs. shHCN2 tumor tissues (GAPDH = loading control); ** *p* < 0.01, *** *p* < 0.001.

To further validate the role of HCN2 in regulating bladder cancer progression, we established xenograft tumor models by subcutaneously injecting HCN2-knockdown (shHCN2) and control (shCtrl) T24 cells into nude mice (*n* = 6 mice/group, with three independent experiments). Tumor growth was monitored, and tumor volume changes revealed that HCN2 knockdown reduced tumor growth (Figure 5A; error bars = mean ± SD). Similarly, tumor weight after sacrifice and tumor excision reflected this trend (Figure 5B; mean ± SD). These results indicate that HCN2 knockdown weakens the tumor growth-promoting effect. IHC staining of tumor tissue sections also showed reduced HCN2 expression in the knockdown group (Figure 5C). Additionally, Western blot analysis showed upregulation of the ferroptosis marker GPX4 in the HCN2-knockdown group, confirming previous findings (Figure 5D). In summary, our in vivo experiments (three independent cohorts) validated that HCN2 knockdown attenuates the tumorigenic potential of bladder cancer cells.

## 3. Discussion

**Figure 6:**
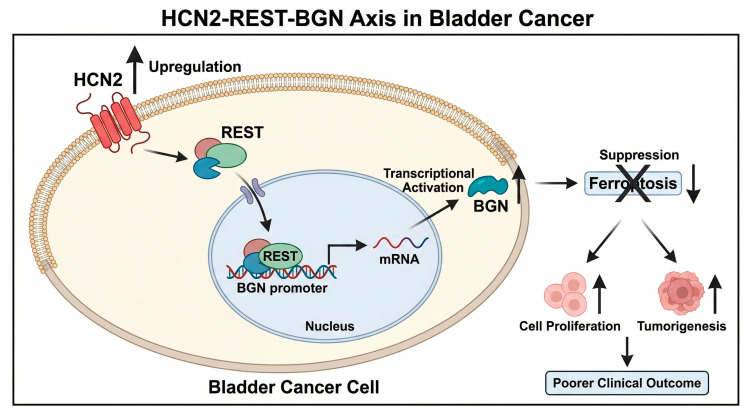
Schematic representation of the HCN2-REST-BGN axis regulating ferroptosis in bladder cancer. 1. HCN2 Upregulation: The red transmembrane—structured HCN2 in the diagram is marked with an “Upregulation” arrow, signifying that HCN2 is notably overexpressed in bladder cancer tissues. 2. HCN2—Induced REST Nuclear Translocation: The arrow pointing from HCN2 to REST indicates that the overexpressed HCN2 facilitates the translocation of the REST transcription factor into the nucleus. 3. REST—Mediated Transcriptional Activation of BGN: Inside the nucleus, REST binds to the BGN gene promoter. The arrow from this REST-BGN promoter complex to mRNA represents the transcriptional activation of the BGN gene, which leads to the synthesis of BGN mRNA. This mRNA is then translated into the BGN protein (shown outside the nucleus). 4. BGN—Driven Ferroptosis Suppression: The “Suppression” notation next to the crossed—out “Ferroptosis” box, along with the downward arrow, demonstrates that the BGN protein inhibits ferroptosis. 5. Oncogenic Outcomes of Ferroptosis Inhibition: When ferroptosis is suppressed, it fuels two key cancer-promoting processes: “Cell Proliferation” and “Tumorigenesis”, as shown by the upward arrows. These processes eventually result in a “Poorer Clinical Outcome”, which is consistent with the abstract's statement that HCN2 overexpression is predictive of a poorer clinical outcome. This diagram visually integrates the experimental findings from bioinformatics, molecular biology assays, and in vitro/in vivo models, highlighting how HCN2 orchestrates a transcriptional program via REST to modulate BGN—mediated ferroptosis and ultimately drive bladder cancer malignancy.

In the present study, we identify HCN2 as a critical regulator of bladder cancer progression and demonstrate that it functions through a previously uncharacterized HCN2-REST-BGN signaling axis that modulates ferroptosis. These findings extend the current understanding of ion channel involvement in tumor biology and suggest that HCN2 represents a potential therapeutic target in bladder cancer, a malignancy with high recurrence rates and limited targeted treatment options (Figure 6).

HCN channels are classically known for their roles in controlling membrane excitability in neurons and cardiomyocytes [16,17]. However, increasing evidence indicates that these channels also contribute to tumorigenesis. In addition to regulating ion flux, HCN family members have been implicated in the modulation of oncogenic signaling pathways, maintenance of cancer stem cell properties, and tumor plasticity [18,19]. In particular, HCN2-mediated membrane depolarization has been associated with enhanced proliferation and stemness in glioma and breast cancer models, suggesting that its bioelectric properties may contribute to tumor progression independently of canonical signaling pathways [18].

A notable aspect of our findings is the isoform-specific role of HCN2. Despite the structural homology among HCN1–4 [20], only HCN2 was found to interact with the transcription factor REST and promote BGN transcription. Analysis of TCGA datasets revealed no significant association between HCN1 expression and BGN levels or patient survival in bladder cancer (Figure S2). Consistently, co-immunoprecipitation assays confirmed that HCN2, but not HCN1 or HCN4, directly binds to REST. This specificity suggests that selective targeting of HCN2 may reduce potential off-target effects on other HCN isoforms. Further studies using isoform-selective inhibitors, such as ZD7288 [21], are required to validate this hypothesis and to determine the contribution of HCN2-mediated bioelectric signaling to bladder cancer progression.

Mechanistically, our data demonstrates that HCN2 promotes tumor progression by suppressing ferroptosis through activation of the REST-BGN pathway. Ferroptosis is an iron-dependent form of regulated cell death characterized by lipid peroxidation and is increasingly recognized as a tumor-suppressive process [14,22]. We show that HCN2 upregulates BGN via REST-dependent transcriptional regulation. In turn, BGN inhibits ferroptosis by reducing lipid peroxidation and maintaining cellular redox balance. These findings are consistent with previous reports implicating BGN in ferroptosis regulation in other cancer types [15,23,24], although its role in bladder cancer has not previously been described.

The involvement of REST in this axis is of particular interest. REST is a transcriptional repressor that silences neuronal genes in non-neuronal tissues, and its dysregulation has been associated with cancer development [25,26,27]. In this study, we provide evidence that HCN2 interacts with REST to attenuate its repressive function on BGN, thereby establishing a regulatory loop that enhances BGN expression. This mechanism may explain the observed resistance to ferroptosis and increased invasive potential in tumors with high HCN2 expression.

From a therapeutic perspective, the HCN2-REST-BGN axis represents a potential vulnerability in bladder cancer. HCN2 was found to regulate apoptosis-inducing factor (AIF)-mediated apoptosis in lung cancer cells and primary cortical neurons, where HCN2 downregulation prevents drug-induced Ca^2+^ increase and subsequent apoptosis [12], and may be applicable in this context. In addition, combining HCN2 inhibition with ferroptosis inducers (e.g., erastin) may provide synergistic effects by simultaneously suppressing pro-survival signaling and promoting cell death. The expression levels of HCN2 and BGN may also serve as potential biomarkers for patient stratification, enabling a more personalized therapeutic approach. Furthermore, combination strategies integrating HCN2 inhibitors with chemotherapy or immunotherapy may help overcome treatment resistance, as suggested by previous studies demonstrating enhanced oxidative stress and apoptosis following HCN2 inhibition [28].

Nevertheless, several limitations should be considered. First, although we demonstrate that BGN mediates the effects of HCN2 on ferroptosis, the downstream molecular mechanisms, particularly those related to lipid metabolism, remain to be fully elucidated. Second, the use of subcutaneous xenograft models does not fully recapitulate the complexity of the tumor microenvironment in bladder cancer. Third, the precise mechanism by which HCN2 regulates REST activity requires further investigation, including structural and biochemical analyses.

Future studies should focus on elucidating the broader regulatory network of the HCN2-REST-BGN axis using approaches such as mass spectrometry and chromatin immunoprecipitation sequencing. In addition, the therapeutic potential of targeting this pathway should be evaluated in more clinically relevant models, including orthotopic and patient-derived systems. Finally, large-scale clinical studies are needed to validate HCN2 and BGN as predictive biomarkers and to assess the efficacy of HCN2-targeted therapies.

## 4. Materials and Methods

### 4.1. Bioinformatics Analysis

RNA sequencing data from bladder urothelial carcinoma and normal tissue samples were downloaded from the TCGA database via the GDC portal. The count data were normalized, and differential gene expression analysis was performed using the R package DESeq2 (version 1.38.3). Additionally, RNA sequencing data of bladder urothelial carcinoma samples from the TCGA database were obtained from cBioPortal (Memorial Sloan Kettering Cancer Center, New York, NY, USA). The clinical information was preliminarily cleaned and organized, followed by prognostic analysis.

### 4.2. Clinical Data and Immunohistochemistry (IHC)

This study was approved by the Ethics Committee of Peking University Cancer Hospital. Normal bladder tissues (*n* = 18) and bladder cancer tissues (*n* = 63) were collected from patients who underwent surgical resection at the hospital between May 2019 and March 2022. All tissue samples were obtained post-cancer resection. Clinical data including age, gender, histological type, degree of differentiation, and lymph node metastasis of bladder cancer were extracted from the surgical pathology archives of the hospital. The tumor status was assessed through clinicopathological diagnosis by the hospital’s clinical pathologists, following specific inclusion and exclusion criteria.

Bladder normal and cancer tissue samples were fixed in 4% neutral-buffered formaldehyde (Servicebio, G1101, Wuhan, China) in phosphate-buffered saline (PBS) and embedded in paraffin. The sections (4 µm) were deparaffinized, rehydrated, and incubated in 0.01 M citrate buffer (pH 6.0, Solarbio, C1030, Beijing, China) at 95 °C for 20 min for antigen retrieval. Endogenous peroxidase activity was blocked with hydrogen peroxide (ZSGB-Bio, PV-9000, Beijing, China), and nonspecific antigens were blocked with 10% normal goat serum (ZSGB-Bio). The sections were then incubated with the primary antibody at 4 °C for 12 h. The primary antibody used was HCN2 (Proteintech, 15057-1-AP, 1:100, Proteintech Group, Inc., Rosemont, IL, USA). The sections were washed with PBS (three times for 5 min each) and then incubated with a goat anti-rabbit secondary antibody (ZSGB-Bio, Beijing, China). The antigen was visualized using a 3,3′-diaminobenzidine (DAB, ZSGB-Bio, Beijing, China) substrate, and the sections were counterstained with hematoxylin (Beyotime Biotech, Inc., Shanghai, China) at 25 °C for 2 min. For the negative control, the primary antibody was replaced with normal goat serum (ZSGB-Bio, Beijing, China). Staining intensity and extent were quantified using the H-score method: H-score = Σ(intensity score × percentage of positive cells), where intensity was graded as 0 (negative), 1 (weak), 2 (moderate), or 3 (strong). Two experienced pathologists independently evaluated the images and staining.

### 4.3. Cell Culture and Transfection

All cell lines used in this study were commercially purchased and not newly derived from patient samples. These included normal bladder epithelial cells (HCV29) and bladder cancer cell lines (TCCSUP, 5637, T24, J82, and RT4), all of which were maintained in our laboratory at Peking University Cancer Hospital. Prior to their utilization, these cell lines underwent rigorous authentication through short tandem repeat (STR) analysis to ensure no mycoplasma contamination. To preserve the cells viability and genomic stability, we adhered to a strict protocol, limiting the use of cells to no more than 15 passages post-thaw. All cell lines were cultured at 37 °C with 5% CO_2_ in RPMI 1640 medium supplemented with 10% fetal bovine serum.

Utilizing RNA interference protocols, we designed target sequences for the gene of interest, synthesized corresponding single-stranded DNA oligos, and cloned them into a linearized vector to create recombinant constructs. Target gene fragments were amplified by PCR with gene-specific primers and integrated into the recombinant vector. Co-transfection of this vector with a GFP-tagged lentiviral vector into 293T cells yielded lentiviruses capable of delivering the interference sequences. Bladder cancer cells (5 × 10^5^, 5637 and T24) were infected with the lentivirus at an MOI of 1 × 10^8^ TU/mL for 20 h, followed by culture continuation post-medium exchange for 72 h. Infected cells were examined for GFP expression and infection efficiency under a fluorescence microscope (IX73, Olympus Corporation, Tokyo, Japan). Sequences for gene silencing are provided in Supplementary Table S1.

### 4.4. qRT-PCR Analysis

Total RNA was extracted using TRIzol reagent, and the RNA purity and concentration were assessed. cDNA was synthesized from the extracted RNA using the Hiscript QRT supermix for qPCR (+gDNA WIPER) kit (Vazyme Biotech Co., Ltd., Nanjing, China). The PCR amplification was performed, and after the reaction, the relative expression levels of genes were calculated using the 2^−ΔΔCt^ method with GAPDH as an internal control. Primer sequences were listed in the Supplementary Table S2.

### 4.5. Western Blot and Co-Immunoprecipitation (Co-IP)

Total proteins were extracted using a cell lysis buffer (Beyotime Biotech, Inc., P0013, Shanghai, China) and were utilized for Western blot and immunoprecipitation (IP) assays. Protein concentrations were determined with a BCA protein assay kit (Beyotime Biotech, Inc., P0009, Shanghai, China). Equal amounts of protein (20 µg per lane) were separated by a 10% SDS-PAGE gel and then transferred onto a polyvinylidene fluoride (PVDF) membrane. The PVDF membrane was blocked with a blocking solution containing 5% non-fat milk in TBST (Solarbio Science & Technology Co., Ltd., T1082, Beijing, China) for 1 h at room temperature on a shaker. Primary antibodies were diluted in the blocking solution and incubated with the membrane for 2 h at room temperature or overnight at 4 °C. After incubation with the corresponding horseradish peroxidase (HRP)-conjugated secondary antibodies diluted in the blocking solution for 1 h at room temperature, the membrane was washed three times with TBST (Solarbio, T1082). Chemiluminescence was detected with the Millipore ECL substrate (MilliporeSigma, Burlington, MA, USA), with GAPDH as a control.

Cell lysates of 5637 and T24 cells, prepared with RIPA buffer, were incubated with anti-REST and anti-HCN2 antibodies (Proteintech Group, Inc., Rosemont, IL, USA) or normal IgG, followed by incubation with protein A/G agarose beads at 4 °C. After centrifugation at 2500 rpm and supernatant removal, the beads were washed and eluted in 2× SDS-PAGE buffer. The samples were then analyzed by Western Blot to evaluate protein interactions via Co-IP. Antibodies used in IHC and Western blotting were listed in the Supplementary Table S3.

### 4.6. Cell Proliferation Assays

For the CCK-8 assay, tumor cells from each group were collected and seeded into 96-well plates at a density of 4 × 10^4^ cells per well. Cell proliferation was assessed using the CCK-8 kit at 24, 48, 72, 96, and 120 h.

For the colony formation assay, cells in the logarithmic growth phase were seeded into 6-well plates (100–300 cells per well) and cultured for 8 days. The cell colonies were fixed with 4% formaldehyde and stained with Giemsa dye. Colonies were photographed under a fluorescence microscope and the number of colonies (each colony containing more than 50 cells) was counted.

### 4.7. Flow Cytometry

Cell pellets were harvested after treatment, washed twice with ice-cold PBS (Gibco, Thermo Fisher Scientific, 10010023, Grand Island, NY, USA), and resuspended in 1× Annexin V Binding Buffer (BD Biosciences, 556454, San Jose, CA, USA) at a density of 1 × 10^6^ cells/mL. Aliquots of 1–2 × 10^5^ cells were stained with Annexin V-FITC (BD Biosciences, 556420; 1:20 dilution) and propidium iodide (PI; Sigma-Aldrich, P4170, 1 μg/mL, St. Louis, MO, USA) for 15–20 min at room temperature in the dark. Staining was quenched by adding 300 μL of 1× Binding Buffer, and samples were analyzed within 1 h using a BD FACSCanto II flow cytometer (BD Biosciences, San Jose, CA, USA) equipped with a 488 nm laser. Data acquisition and analysis were performed using FlowJo v10.8.1 software (Tree Star, Inc., Ashland, OR, USA). Quadrants were defined as follows: Q1 (Annexin V^−^/PI^+^): necrotic cells; Q2 (Annexin V^+^/PI^+^): late apoptotic cells; Q3 (Annexin V^+^/PI^−^): early apoptotic cells; Q4 (Annexin V^−^/PI^−^): viable cells. Apoptosis rates were calculated as the sum of Q2 and Q3.

### 4.8. Scratch Assay

Transfected or treated cells were seeded into 24-well plates. When cell confluence reached 90%, a wound was created on the cell monolayer using a 10 μL pipette tip. The remaining cells were washed three times with 1× PBS and incubated in serum-free medium for 24 h. Images were acquired at 0 and 24 h using a microscope (Olympus Corporation, Tokyo, Japan). Migration rate (%) was calculated as [(A_0_ − A_24_)/A_0_] × 100%, where A_0_ and A_24_ represent wound areas at 0 and 24 h, respectively, measured with ImageJ v1.53t software (National Institutes of Health, Bethesda, MD, USA).

### 4.9. Transwell Invasion/Migration Assay

Cell culture medium containing 20% fetal bovine serum (FBS) was added to the lower chamber of the Transwell insert (pore size 8 μm; Costar, Corning Inc., Corning, NY, USA). A total of 6–8 × 10^4^ transfected or treated cells were resuspended in 200 μL of serum-free DMEM and added to the upper chamber. After 48 h of incubation, cells were fixed with methanol for 15 min and stained with 0.5% crystal violet at room temperature for 20 min. Finally, the cells were imaged and counted using a microscope.

### 4.10. Chromatin Immunoprecipitation (ChIP)-qPCR

ChIP was performed using the SimpleChIP^®^ Enzymatic Chromatin IP Kit (Cell Signaling Technology, Inc., Danvers, MA, USA) following the manufacturer’s instructions. After cross-linking, chromatin was digested with micrococcal nuclease to generate ~150–900 bp fragments. REST antibody (Proteintech Group, Inc., 22242-1-AP, Rosemont, IL, USA) and control IgG (Cell Signaling Technology, Inc., 2729, Danvers, MA, USA) were used for immunoprecipitation. After four washes and reversal of cross-links, precipitated DNA was analyzed by qRT-PCR to assess REST-DNA interactions.

### 4.11. Animal Experiments

Twelve female BALB/c nude mice (4 weeks old; Jiangsu Jicui Yaokang Biotechnology Co., Ltd., Nanjing, China) were maintained under specific pathogen-free conditions. The mice were randomly divided into two groups: shCtrl and shHCN2. T24 bladder cancer cells were injected subcutaneously into the right flank of the mice, and tumor volume and body weight were monitored. Tumor growth was measured at designated time points (days 3, 5, 7, 10, and 12) using calipers, and tumor volume was calculated using the formula π/6 × L × W^2^, where L is the tumor length and W is the tumor width. The mice were sacrificed 12 days after injection, and the xenograft tumors were weighed. The excised tissues were subjected to subsequent immunohistochemical analysis.

### 4.12. Statistics

Data are presented as mean ± SD from at least three independent experiments. Statistical analyses were performed using GraphPad Prism 9 (GraphPad Software, LLC, La Jolla, CA, USA) and SPSS 27.0 (IBM Corp., Armonk, NY, USA). Depending on data type, Student’s *t*-test, chi-square test, Mann–Whitney U test, and one-way ANOVA were used. Kaplan–Meier survival analysis was performed and compared by the log-rank test. A *p* value < 0.05 was considered statistically significant.

## 5. Conclusions

Overall, our findings identify the HCN2-REST-BGN axis as a key regulator of bladder cancer progression through modulation of ferroptosis. These results provide mechanistic insight into the role of ion channels in cancer and support the development of HCN2-targeted therapeutic strategies.

## Data Availability

The original contributions presented in this study are included in the article/Supplementary Materials. Further inquiries can be directed to the corresponding authors.

## Supplementary Materials

The following supporting information can be downloaded at: https://www.mdpi.com/article/10.3390/ijms27083433/s1.
