# Supplementary material for: HCN2 Promotes BGN Transcription via REST to Regulate Ferroptosis and Tumor Progression in Bladder Cancer
Source: Int J Mol Sci. 2026 Apr 11;27(8):3433. doi: 10.3390/ijms27083433 (PMC13116553; doi:10.3390/ijms27083433)
Supplement: Supplementary file 1 [file ijms-27-03433-s001.zip › ijms-4180666-supplementary.pdf]

Table S1. The target sequences.

Gene	No.	Target sequence (5'-3')
HCN2	17432-KD-1	CGGCATTGTGATCGAGGACAA
	17432-KD-2	GGCCATGCTGACCAAGCTCAA
	17432-KD-3	AAGGAGATGAAGCTGTCCGAT
BGN	21062-KD-1	CCTGAATGAACTCCACCTAGA
	21063-KD-2	GAGAACAGTGGCTTTGAACCT
	21064-KD-3	CGGCATCAGCCTCTTCAACAA

Table S2. Primers used in qPCR and ChIP-qPCR.

Gene	Forward primer sequence (5'-3')	Reverse primer sequence (5'-3')
HCN2	TCCGCTACATCCATCAGTG	CAGCATAGGCACCAGGAA
BGN	GAGACCCTGAATGAACTCCACC	CTCCCGTTCTCGATCATCCTG
GAPDH	TGACTTCAACAGCGACACCCA	CACCCTGTTGCTGTAGCCAAA

Table S3. Antibodies used in western blotting and IHC.

Primary antibodies	Dilution in WB	Source species	Company	Catalog No.
HCN2	1:1000	Rabbit	Proteintech	55245-1-AP
GAPDH	1:30000	Mouse	Proteintech	60004-1-lg
REST	1:50/1:1000	Rabbit	Proteintech	22242-1-AP
BGN	1:1000	Rabbit	Affinity	DF8189
GPX4	1:2000	Rabbit	Boster	BM5231
SLC7A11	1:1000	Rabbit	Boster	A03036-2
ACSL4	1:2000	Rabbit	Proteintech	22401-1-AP
TFR1	1:1000	Rabbit	Boster	BM4886
Histone H3	1:2000	Rabbit	CST	4499S
**Primary antibodies**	**Dilution in IHC**	**Source species**	**Company**	**Catalog No.**
HCN2	1:100	Rabbit	proteintech	55245-1-AP
Ki67	1:200	Rabbit	abcam	ab16667
Secondary antibody	Dilution	-	Company	Catalog No.
HRP Goat Anti-Rabbit IgG (WB)	1:3000	Rabbit	Beyotime	A0208
HRP Goat Anti-Mouse IgG (WB)	1:3000	Mouse	Beyotime	A0216
HRP Goat Anti-Rabbit IgG (IHC)	1:200	Rabbit	Abcam	Ab97080

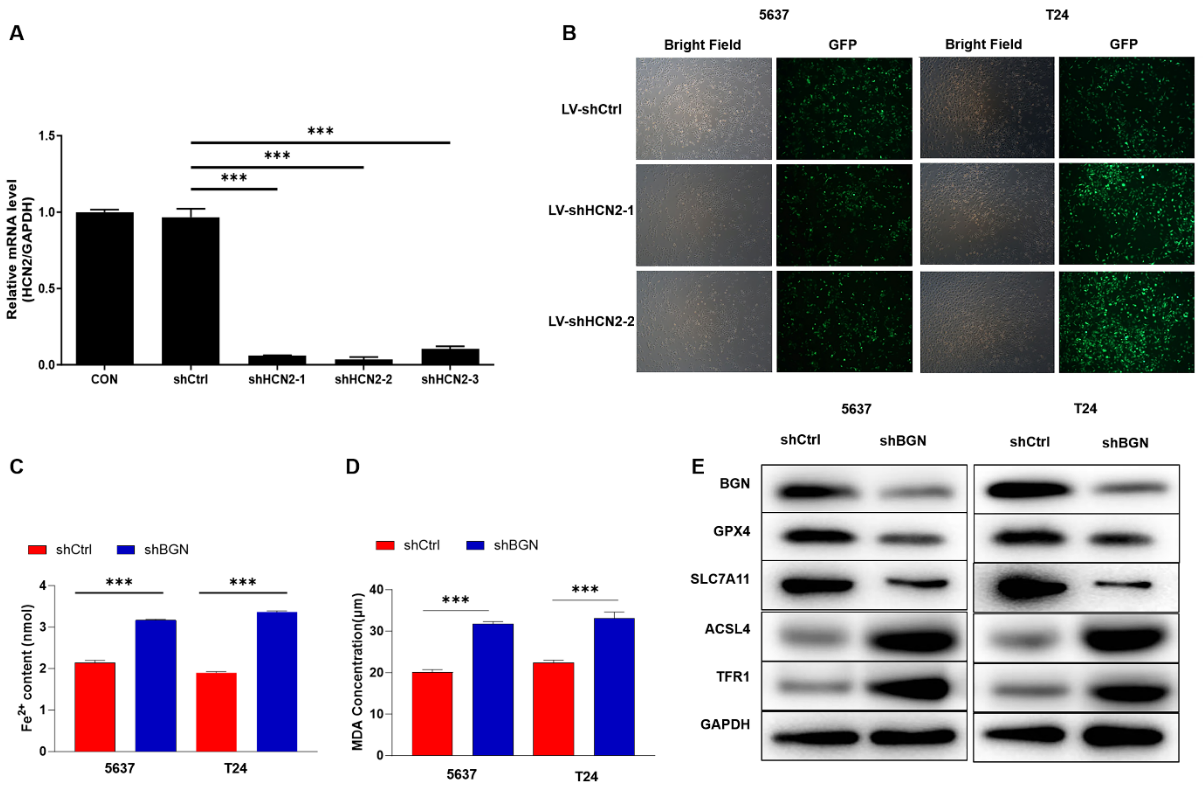

Figure S1. Evaluation of infection efficiency and ferroptosis in bladder cancer cell model. A. Efficiency of HCN2 knockdown in bladder cancer cells by lentiviral infection assessed by qRT-PCR. **B.** Efficiency of lentiviral infection and cellular condition in 5637 and T24 cells post-infection with shCtrl, shHCN2-1, and shHCN2-2. **C-E.** In 5637 and T24 cells with BGN knockdown and their respective controls, the promotion of ferroptosis in bladder cancer cells by BGN knockdown was demonstrated through the detection of iron content (C), MDA levels (D), and the expression of ferroptosis-related proteins (GPX4, SLC7A11, ACSL4, TFR1) (E).

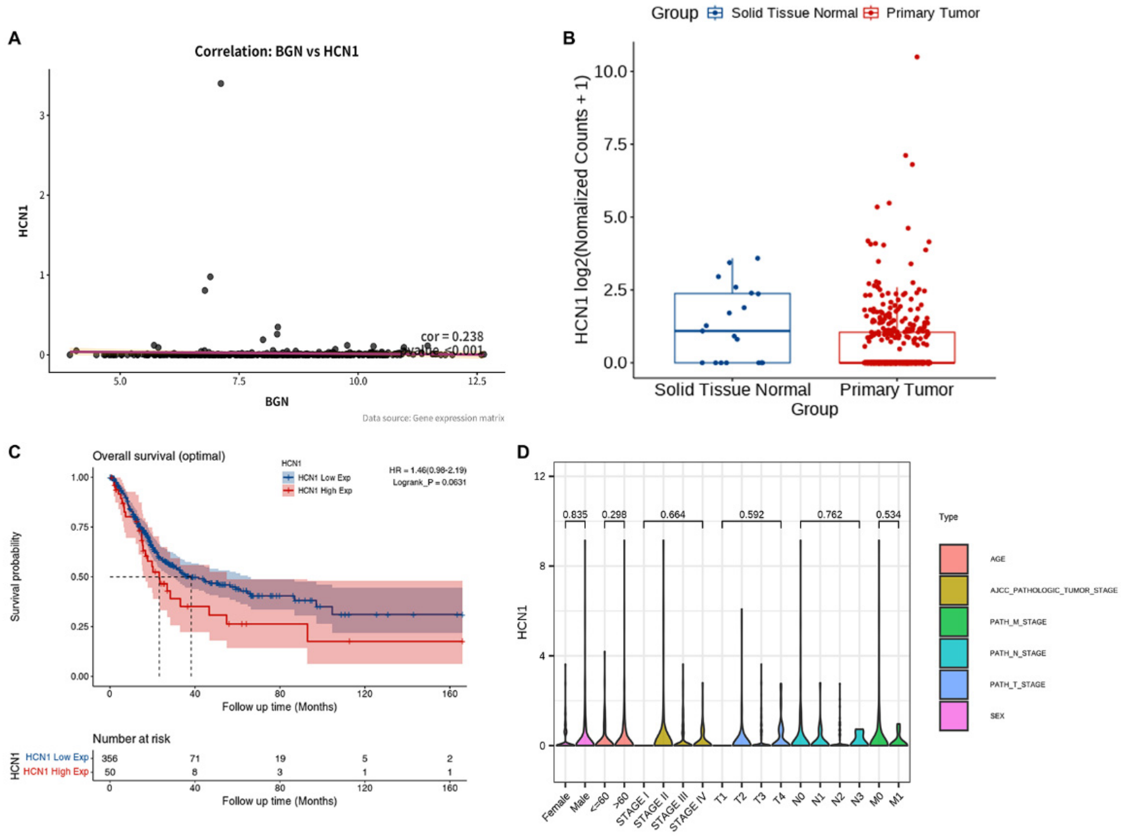

Figure S2. HCN1 expression in bladder cancer. A. Correlation between HCN1 and BGN expression in bladder cancer as determined by Pearson Correlation Analysis using TCGA Database. **B.** A box plot was used to visualize the expression of HCN1 between bladder urothelial carcinoma and normal urothelial tissues, based on RNA-seq data retrieved from TCGA database. **C.** The expression level of HCN1 across all bladder urothelial carcinoma samples was used as the cutoff value to dichotomize patients into high- and low-expression groups. Overall survival (OS) differences between the two groups were assessed using the log-rank test. **D.** Relationship between HCN1 expression and tumor characteristics in patients with bladder cancer.
